# Supplementary material for: Facilitators and barriers of infectious diseases surveillance activities: lessons from the Global Polio Eradication Initiative - a mixed-methods study
Source: BMJ Open. 2022 May 12;12(5):e060885. doi: 10.1136/bmjopen-2022-060885 (PMC9109099; doi:10.1136/bmjopen-2022-060885)

Supplemental Table 1: Definitions of internal factors for successful surveillance and challenges in the implementation of surveillance activities

Factors	Definitions
**Internal factors for surveillance success**	
Implementation process	Successful surveillance due to how the activity was implemented, including the planning, execution strategies, reflection and evaluation of activities, or adjustments made to the plan.
Individual attributes	Surveillance success due to the characteristics of individuals within your organization involved in polio eradication activities including individual knowledge, self-efficacy, stage of change, perception, and other personal attributes.
Organizational settings	Surveillance success due to factors related to your organization supporting the polio eradication program
Polio program characteristics	Successful surveillance due to activity that was used towards eradicating polio, including technologies that were adopted by the organization/individual implementing the activities
Successful surveillance activity	Defined as respondent’s perceived accomplishment of the primary objective for implementing surveillance activities
**Surveillance challenges**	
Organizational challenges	Challenges related to characteristics of organization supporting the polio eradication program including structural characteristics, networks, culture, implementation climate, and readiness.
GPEI program challenges	Challenges related to the activity that was used towards eradicating polio including intervention source, strength and quality of evidence, relative advantage, adaptability, complexity, design quality and packaging, and cost.
External challenges	Challenges related to political, economic, social, technological or environmental settings.

Supplemental Table 2: Distribution of sub-domains of surveillance challenges stratified by the internal factor for successful surveillance

	Context of internal factors for successful Surveillance		
**Challenges**	**Implementation Process (N=511)**	**Others (N=291)**	**P-value**
**Organizational challenges, n (%)**			
Structural Characteristics	11 (12.94)	7 (19.44)	0.358
Networks	38 (44.71)	19 (52.78)	0.416
Culture	26 (30.59)	13 (36.11)	0.552
Implementation Climate	46 (54.12)	13 (36.11)	0.070
Implementation Readiness	45 (52.94)	23 (63.89)	0.267
Other personal attributes	4 (4.71)	1 (2.78)	0.626
**GPEI Program challenges, n (%)**			
Intervention Source	23 (46.00)	15 (34.09)	0.240
Evidence	21 (42.00)	14 (31.82)	0.308
GPEI Advantage	11 (22.00)	7 (15.91)	0.454
Adaptability to Local Context	26 (52.00)	19 (43.18)	0.393
Trialability	6 (12.00)	7 (15.91)	0.584
Complexity	22 (44.00)	18 (40.91)	0.762
Design	11 (22.00)	5 (11.36)	0.171
Cost	28 (56.00)	18 (40.91)	0.144
Others	5 (10.00)	0 (0.00)	**0.031**
**External challenges, n (%)**			
Political	67 (25.77)	49 (34.51)	0.065
Economic	95 (36.54)	47 (33.10)	0.490
Social	115 (44.23)	53 (37.32)	0.180
Technological	60 (23.08)	41 (28.87)	0.200
Others[a]	78 (30.00)	25 (17.61)	**0.007**

Only “yes” are shown in the table

[a]Others defined as environment where activity implemented was prohibitive and did not contribute to the success of polio eradication, including the global climate and ineffective cross-organizational collaboration.

Supplemental Figure 1: Flow chart of study population

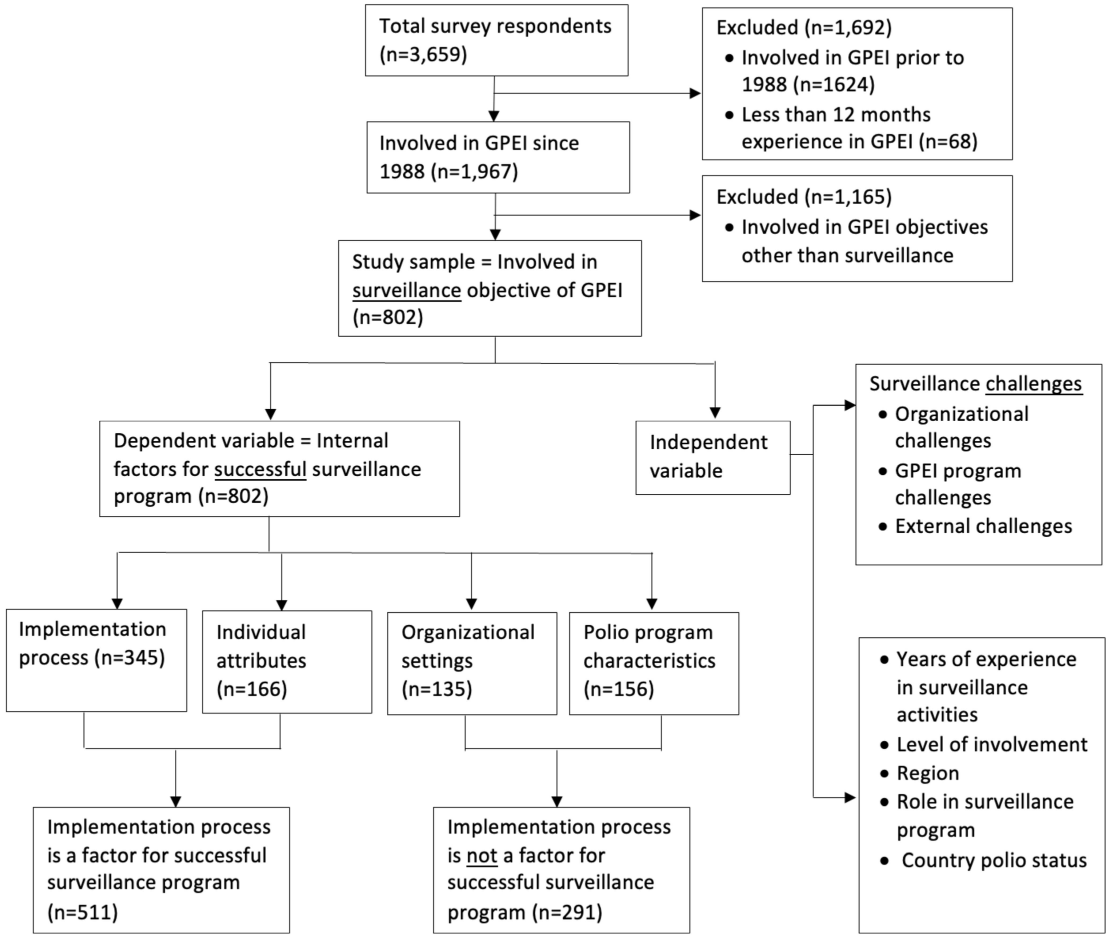

Supplement: Supplementary data [file bmjopen-2022-060885supp001.pdf]
